# Propoxur resistance associated with insensitivity of acetylcholinesterase (AChE) in the housefly, *Musca domestica* (Diptera: Muscidae)

**DOI:** 10.1038/s41598-020-65242-3

**Published:** 2020-05-21

**Authors:** Chunmei You, Chao Shan, Juanjuan Xin, Jing Li, Zhuo Ma, Yi Zhang, Xiaopeng Zeng, Xiwu Gao

**Affiliations:** 1 Department of Entomology, China Agricultural University, Beijing, 100193 China; 2 Technology Center of Qinhuangdao Customs District, Qinhuangdao, 066004 China; 3 Haidian District Center for Disease Prevention and Control, Beijing, 100094 China; 4 Beijing Center for Diseases Control and Prevention, Beijing, 100013 China; 5 Dongcheng District Center for Diseases Prevention and Control, Beijing, 100009 China

**Keywords:** Enzymes, Entomology

## Abstract

Two unique housefly strains, PSS and N-PRS (near-isogenic line with the PSS), were used to clarify the mechanisms associated with propoxur resistance in the housefly, *Musca domestica*. The propoxur-selected resistant (N-PRS) strain exhibited >1035-fold resistance to propoxur and 1.70-, 12.06-, 4.28-, 57.76-, and 57.54-fold cross-resistance to beta-cypermethrin, deltamethrin, bifenthrin, phoxim, and azamethiphos, respectively, compared to the susceptible (PSS) strain. We purified acetylcholinesterase (AChE) from the N-PRS and PSS strains using a procainamide affinity column and characterized the AChE. The sensitivity of AChE to propoxur based on the bimolecular rate constant (K_i_) was approximately 100-fold higher in the PSS strain compared to the N-PRS strain. The cDNA encoding *Mdace* from both the N-PRS strain and the PSS strain were cloned and sequenced using RT-PCR. The cDNA was 2073 nucleotides long and encoded a protein of 691 amino acids. A total of four single nucleotide polymorphisms (SNPs), I162M, V260L, G342A, and F407Y, were present in the region of the active site of AChE from the N-PRS strain. The transcription level and DNA copy number of *Mdace* were significantly higher in the resistant strain than in the susceptible strain. These results indicated that mutations combined with the up-regulation of *Mdace* might be essential in the housefly resistance to propoxur.

## Introduction

The housefly, *Musca domestica*, is found in the vicinity of human habitations throughout the world. It is a serious threat to human and animal health because it carries bacteria and protozoans that can cause many serious diseases^1^. For decades, housefly control has been dependent on the application of insecticides, including organophosphate compounds (OPs), carbamate compounds (CBs), pyrethroids and so on. Propoxur, a carbamate insecticide, was widely used against houseflies due to its long-lasting and broad-spectrum efficacy^2^. Unfortunately, resistance to propoxur in houseflies has been detected all over the world^3,4^^.^ At present, resistant houseflies have been identified in 21 provinces in China^5^. In Beijing, the resistance level of the housefly to propoxur was higher than to other insecticides, like DDVP, chlorpyrifos, beta-cypermethrin, and deltamethrin^6^. A housefly population that was collected from a field in Golmud city, Qinghai province, China, has developed more than 1220-fold resistance to propoxur relative to the laboratory susceptible strain^7^.

Acetylcholinesterase (AChE, EC 3.1.1.7), the target of OPs and CBs, is a serine esterase in the α/β fold hydrolase enzyme family that terminates nerve impulses at cholinergic synapses by breaking down the neurotransmitter acetylcholine (ACh)^8^. AChE is encoded by two distinct genes (*ace-1* and *ace-2*) in insects^9^. Usually quantitative and qualitative changes in AChE are associated with insecticide resistance^10^. AChE insensitivity caused by various point mutations is one of the major resistance mechanisms in many arthropods^11^. Only one locus, *ace-2*, encoding *Mdace* has been reported in *M*. *domestica*^12^. Six widespread point mutations (I162M, V260L, G342A, G342V, F407Y, and G445A) in and around the catalytic site have been identified in many strains (including 49R, CH2, 77M, 690ab, and YBOL) resistant to OPs and CBs^13,14^. These mutations have been discussed in detail in previous studies, including dimensional structure modeling and site-directed mutagenesis^13,14^. Some additional mutations of *Mdace* at different sites were reported in other housefly strains^15–17^.

Modifications of *ace-2* associated with resistance have been described in other resistant insects such as *Drosophila melanogaster*^18^, *Lucilia cuprina*^19^, and *Bactrocera oleae*^20^. Many mosquito species have two distinct *ace* genes, and mutation (G119S of the *ace-1* gene) in the oxyanion hole of the enzyme confers high resistance to OPs and CBs in *Culex pipiens*^21^, *Anopheles gambiae*^22^, and *An*. *albimanus*^23^.

Overproduction of *ace* (gene duplication and up-regulation) as an evolutionary response to OP and CB selection pressure has been reported in several arthropod species. In *An*. *gambiae*, the *ace-1* gene was systematically duplicated in all 173 resistant individuals among 398 mosquitoes tested by resolving the genomic structure of the duplications to design a diagnostic test for duplication^24^. In *D. melanogaster*, it has been verified that the increased amount of AChE is correlated with resistance to parathion in some field populations collected from 66 sites^25^. In *Aphis gossypii*, the relative mRNA and DNA expression of *ace-2* were both significantly higher in the omethoate resistant strain compared to the susceptible strain^26^. Extensive gene duplication of *ace* associated with OP resistance was also studied in *Tetranychus urtica*^27–29^ and *Blattella germanica*^30^. However, there is little research about how overexpression of *Mdace* is involved in propoxur resistance in the housefly. In previous research, we established a near-isogeneic line (NIL) of housefly with propoxur resistance to study the inheritance pattern of resistance^31^. In this paper, we first investigated the cross-resistance to other insecticides and characterized the AChE from resistant (N-PRS) and susceptible (PSS) strains. Subsequently, we reported *Mdace* mutations putatively associated with resistance by comparing the sequences among the resistant and susceptible strains. In addition, we examined the overexpression of the resistant *Mdace* gene further increasing the tolerance ability of the housefly to propoxur. Based on these results, we investigated the involvement of mutations and *Mdace* overexpression in propoxur resistance.

## Results

### Cross-resistance patterns

The N-PRS strain was more than 1035-fold resistant to propoxur compared to the PSS strain (Table 1). The N-PRS strain developed a high level of cross-resistance to phoxim (57.76-fold) and azamethiphos (57.54-fold). In the N-PRS strain, the resistance was 12.06-fold to deltamethrin and 4.98-fold to bifenthrin. However, the resistance was only 1.70-fold compared with the PSS strain for beta-cypermethrin.

**Table 1:** Toxicity of insecticides to PSS and N-PRS strains.

Insecticide	Strain	N^a^	LD_50_ (95%FL) (ng/fly)	Slope ± SE	RR^b^
Propoxur	PSS	360	190.55 (145.39–238.11)	1.54 ± 0.35	
	N-PRS	360	>197,400		>1035
Beta-cypermethrin	PSS	360	74.72 (63.20–83.25)	5.21 ± 1.04	
	N-PRS	360	126.71 (113.37–138.68)	8.56 ± 1.20	1.70
Deltamethrin	PSS	360	3.95 (1.57–9.86)	2.47 ± 0.34	
	N-PRS	360	47.64 (34.78–61.17)	3.87 ± 0.46	12.06
Bifenthrin	PSS	360	20.61 (11.12–30.70)	1.73 ± 0.34	
	N-PRS	360	88.18 (75.09–102.77)	2.95 ± 0.35	4.98
Phoxim	PSS	360	3.16 (1.40–12.10)	1.05 ± 0.18	
	N-PRS	360	208.51 (109.43–258.95)	4.98 ± 0.77	57.76
Azamethiphos	PSS	360	2.82 (1.09–5.61)	3.11 ± 0.47	
	N-PRS	360	162.25 (142.65–180.70)	3.79 ± 0.57	57.54

^a^Number of houseflies used in the bioassay.

^b^RR = LD_50_ of the N-PRS /LD_50_ of the PSS strain.

### Purification and characterization of AChE

Purified AChE was made from both strains by procainamide affinity chromatography (Table 2). The overall purification factors and yields were 361.84-fold and 16.77% for the PSS strain, and 477.27-fold and 15.53% for the N-PRS strain, respectively. The purification factor for the N-PRS strain was not different from the PSS strain. Enzyme activity of crude extract and purified AChE from both strains were measured, and there were no apparent differences between the PSS strain and the N-PRS strain.

**Table 2:** Purification of AChE from houseflies by procainamide-based affinity chromatography.

Strain	PSS		N-PRS	
Procedure	Crude	Affinity chromatography	Crude	Affinity chromatography
Volume (ml)	5.0	1.7	5.0	2.5
Protein (mg/ml)	0.86	0.12	1.11	0.07
Total protein (mg)	4.30	0.20	5.55	0.18
Specific activity(OD_412_/mg·pro·min)	0.19	68.75	0.15	71.59
Total activity (OD_412_/min)	0.82	13.75	0.83	12.89
Yield (%)^a^	100	16.77	100	15.53
Purification factor^b^	1	361.84	1	477.27

^a^Yield (%) = the total activity of the affinity chromatography enzyme/the total activity of the crude enzyme.

^b^Purification factor = the specific activity of the affinity chromatography enzyme/the specific activity of the crude enzyme.

The biomolecular rate constant, K_i_, which provides a good measurement of AChE sensitivity to inhibition by propoxur was approximately 100 times higher in the PSS strain than the N-PRS strain (Table 3). The insensitivity ratio of R to S enzyme to methomyl was 35, and to esterine was 112 using K_i_ evaluation. Moreover, two selected OP compounds were used to compare the sensitivity levels of purified AChE, the N-PRS strain was 64- and 70-fold less sensitive to inhibition by azamethiphos and DDVP, respectively.

**Table 3:** K_i_ of purified AChE from PSS and N-PRS strains in the housefly.

Insecticides	K_i_ ± SE (×10^−5^mol·min^−1^)		Ratio^a^ (S/R)
	PSS	N-PRS	
Propoxur	77.65 ± 12.02	0.78 ± 0.04	99.6
Methomyl	37.79 ± 10.67	1.08 ± 0.08	35.0
Eserine	390.54 ± 15.34	3.46 ± 0. 98	112.87
Azamethiphos	68.33 ± 3.28	1.07 ± 0.04	63.9
DDVP	55.15 ± 9.45	0.72 ± 0.13	76.6

^a^Ratio = Ki of PSS /Ki of N-PRS.

### Identification of mutations related to propoxur resistance in *Mdace*

The entire coding region of the housefly *Mdace* gene was successfully cloned and sequenced. The deduced *Mdace* sequences of the PSS and N-PRS strains both have 691 amino acids, containing an unusually long 79-residue signal peptide and a 612-residue mature protein sequence, like the YBOL strain^13^. Seven single nucleotide polymorphisms (SNPs), Ile9Thr (I9T), Arg17Trp (R17W), Ser27Tyr (S27Y), Ile162Met (I162M), Val260Leu (V260L), Gly342Ala (G342A), and Phe407Tyr (F407Y) were identified in the resistant houseflies (Table 4). Three mutations (I9T, R17W, S27Y) were identified in the signal peptide, but because this N-terminus hydrophobic peptide is present in the precursor and absent in the mature protein, these amino substitutions may not affect insecticide resistance. We observed that the N-PRS strain carried homozygous resistant alleles at the other four mutations, and the PSS strain carried different homozygous alleles at the 162 amino acid (Table 5).

**Table 4:** Non-synonymous mutations in propoxur resistant housefly.

Nucleotide		Amino acid	
Site	Substitution^a^	Site	Substitution^a^
26	ATA → ACA	9	I → T
49	CGG → TGG	17	R → W
80	TCT → TAT	27	S → Y
486	ATA → ATG	162	I → M
778	GTC → CTC	260	V → L
1025	GGC → GCC	342	G → A
1220	TTT → TAT	407	F → Y

^a^The former nucleotide or amino acid was detected in the PSS strain and the latter in the N-PRS strain.

**Table 5:** Frequency of polymorphisms of amino acid mutation sites in *Mdace*.

Population	I162M ATA → ATG			V260L GTC → CTC			G342A GGC → GCC			F407Y TTT → TAT		
	A/A	A/G	G/G	G/G	G/C	C/C	G/G	G/C	C/C	T/T	T/A	A/A
PSS	60	0	40	100	0	0	100	0	0	100	0	0
N-PRS	0	0	100	0	0	100	0	0	100	0	0	100

### Analysis of *Mdace* gene expression

**Figure 1:**
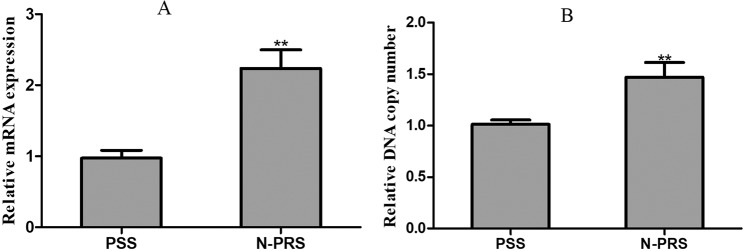
(**A**) Relative mRNA expression level and (**B**) DNA copy number of *Mdace* in the PSS and N-PRS strains. The bars show the standard deviation of the average for three replicates. Student’s *t*-test; **Indicate P < 0.01.

To determine whether *Mdace* gene expression may be related to propoxur resistance, the relative mRNA expression level and DNA copy number in the N-PRS strain and PSS strain were determined by qRT-PCR (Fig. 1). The results showed that the mRNA expression level was significantly up-regulated in the resistant strain compared to the susceptible strain, with the ratio of 2.30 and a p-value of 0.0004. The DNA copy number was 1.61-fold higher in the N-PRS strain than in the PSS strain, with a p-value of 0.0098.

## Discussion

Houseflies have developed resistance to almost all widely used OP and CB insecticides^13,14^. In our previous study, we speculated that there was one main factor associated with propoxur resistance in the N-PRS strain^31^.

In the current study, we noticed that the N-PRS strain possessed the cross-resistance to OPs, which target the same site as CBs. The OPs and CBs resistance are divided into two main classes of target resistance according to the bioassay results and bimolecular rate constant K_i_^32^. The insects with Pattern I are more resistant to CBs than OPs or alternatively the insects with Pattern II are not significantly different in resistance to OPs and CBs and may be especially resistant to OPs^32^.The case of M. *domestica* is identified as Pattern II resistance based on about the same K_i_ values for OPs and CBs^14,32^.

Previously, in a similar study, the propoxur-selected resistant (SH-CBR) strain of housefly exhibited much greater resistance to CBs (propoxur, methomyl, and carbofuran) than OPs (dimethoate, methamidophos, chlorpyrifos, and parathion) both from the bioassay results and bimolecular rate constant (K_i_) ratios, indicating pattern I^33^. However, our results corresponded to Pattern II resistance in the housefly strain. In the present study, we successfully extracted and purified AChE from the PSS strain and the N-PRS strain by procainamide affinity chromatography. Even though the N-PRS strain exhibited higher resistance to CBs than OPs in the bioassay study, the results clearly showed that purified AChE had similar sensitivity to OPs and CBs. The variation was owing to (1) different OPs (DDVP and azamethiphos) used for our study; (2) the AChE that exhibited the insensitivity was purified rather than being a crude extract. Further research is necessary to determine the cause of this inconsistency. In addition, the N-PRS strain developed resistance to some pyrethroids, suggesting the propoxur resistance of the housefly exhibited cross-resistance not only to those insecticides targeting on AChE, but also targeting on different sites. The bioassays with synergists (PBO, DEM and DEF) were used to determine whether metabolic mechanisms involved in propoxur resistance^34^. PBO could significantly increase the toxicity of propoxur to the N-PRS strain, whereas no synergism with DEM and DEF was observed. The enzyme activities of P450s, carboxylesterases and glutathione transferases in the N-PRS strain were not different from that in the PSS strain. Further gene expression of fourteen P450s genes associated with insecticide resistance of two strains demonstrated that ten genes were overexpressed in the N-PRS strain compared to the PSS strain (unpublished data).

Although the lower purification factors and yields associated with structural differences of AChE from the N-PRS strain were not found in our study, the hypothesis on mutations of *Mdace* associated with resistance in the N-PRS strain was supported by lower sensitivity of purified enzyme to different CBs and OPs compared to the PSS strain. This result was in accordance with a study suggesting that lower sensitivity was the major resistance mechanism of the housefly^33^.

The 2073 bp *Mdace* gene encodes a 79 residue signal peptide and a mature protein of 612 amino acids. Our investigation unveiled four, non-synonymous SNP mutations, I162M, V260L, G342A, and F407Y in mature AChE protein, indicating that they were resistant to OPs and CBs. However, I162M was also found in the PSS strain just like the sensitive type of *Mdace* in the Cornell Toyama (CT) strain, indicating it has no effect on the sensitivity to insecticides^13^. Val260 and Leu260 are adjacent to the acyl pocket. Gly342 is directed against the active-site Ser315, occupying the space opposite of the catalytic triad formed by three residues (Ser314, Glu443, and His556)^14^. Phe407 is an important residue that frames the acyl binding pocket, and F407Y modifies the available space within the acyl-binding pocket, which is considered the most important mutation^14^. There were no new substitutions in mature AChE protein identified in our study. Five mutations (P119S, V182L, G265A, F327Y, and D342V) were identified in a resistant housefly strain selected by propoxur from Shanghai, China^33^. A novel mutation, D342V, would probably change the shape of the acyl pocket and explain the decreased affinity of AChE to CBs, not found in our near-isogenic line (NIL) strain obtained through a rotating process of genetic hybridization methods.

Moreover, point mutations in *Mdace* were found in our study, and the experiments indicated that the overexpression of *Mdace* was remarkably higher in the N-PRS strain than in the PSS strain. Clear evidence has shown that the overexpression of *ace* (*ace-1* and *ace-2*) was required to make up the reduced catalytic activity and the possible fitness cost caused by mutations in many insects^16,25–29,35^. Quantitative changes allow living organisms to adapt to changing environments, like consequent insecticide selection pressure^26^. Lee *et al*. speculate that the overexpression of *ace* in the resistant strain improved their resistance to insecticides, perhaps by providing more molecular targets for OPs and CBs^29^. In the N-PRS strain, the increased transcript level of *Mdace* combined with the increased DNA copy numbers relative to the PSS strain, was related to a high insensitivity of the AChE enzyme to propoxur. In previous report on *Tetranychus urticae* Koch, the proportion among the transcription of *Tuace* and the level of duplication were proximately 1:1 in the three strains (UD, PyriF, and AD) examined, suggesting that the actively transcribed all duplicated copies of *Tuace* are associated with transcription of *Tuace*^27^. The quantity of TuAChE and the *Tuace* copy numbers were in the direct ration^28^. The fold differences between mRNA and DNA in our study were probably similar. However, we need further research to determine whether there was a difference between relative mRNA expression level and DNA copy number. Our study indicated that a target-mediated mechanism, which is based on both site mutations and overproduction in target expression, plays a significant role in resistance to propoxur in the housefly strain.

## Materials and methods

### Insects

Two housefly strains were used in this study. The propoxur-susceptible strain (PSS) was obtained from National Taiwan University in 1987, and reared in the laboratory without exposure to any insecticides^36^. The near-isogenic line (NIL) with propoxur resistance (N-PRS) was established by Shan *et al*. in 2016^31^. Two housefly strains were reared under standard laboratory conditions (25 ± 1 °C, 60–80% RH, and a photoperiod of 16:8[light:dark]) and supplied with water, sugar, and milk powder^37^.

### Chemicals

Propoxur (99%) was purchased from Bayer Company Limited. Methomyl (80%) was from Shandong Jining Chemical Plant. Azamethiphos (95%) was from Shanghai Yongyuan Chemical Group Company Limited. Beta-cypermethrin (95.2%) and DDVP (98.7%) were obtained from Tianjin Longdeng Chemical Company Limited. Bifenthrin (95%) and Deltamethrin (99%) were from Jiangsu Yangnong Chemical Company Group Company Limited. Leupeptin, phenylmethanesulfonyl fluoride (PMSF), eserine hemisulfate, procainamide hydrochloride, bovine serum albumin (BSA), and acetylthiocholine iodide (ATChI) were acquired from Sigma Chemical Company. Tetraethylammonium iodide (Net_4_I) was from Sigma-Aldrich Company Limited. EDTA-Na_2_ was from BIO-RAD Laboratories. 2-Ethoxy-1-ethoxycarbonyl-1,2-dihydroquinoline (EEDQ) and 5,5’-dithiobis-(2-nitrobenzoic acid) (DTNB) were from Fluka Chemical Company. ECH-sepharose 4B was from Beijing Solarbio Science & Technology Company Limited. Sephadex G-25 columns were purchased from Amersham Pharmacia Biotech Incorporated. Other chemicals were purchased from commercial suppliers, all were analytical quality.

### Bioassays

The bioassays were performed by a topical application method to assess the level of resistance in four-day-old female houseflies^38^. Insecticides were dissolved in acetone and diluted to 5–7 concentrations that gave a 10%~ 90% mortality rate. The treatment for each concentration was performed three repetitions and each 20 flies were used for each replicate. Control groups were treated with acetone alone. All the treated houseflies were placed in 240 mL plastic jars with a piece of sponge saturated in sugar water. The mortality was assessed at 24 h after treatment. The bioassays data was pooled and calculated using POLO-Plus 2.0 software (LeOra Software Lnc., Berkeley, CA).

### Purification and characterization of AChE

#### Purification of AChE

The purified AChE from both PSS and N-PRS strains were obtained by affinity chromatography using procainamide as the affinity ligand, and the procedure was as follows^39,40^:

Step 1: Preparation of the crude extract. Heads of four-day-old houseflies from each strain were homogenized in ice-cold phosphate buffer (0.1 M pH 7.5, containing 1% Triton, 1 μM leupeptin, 1 mM EDTA-Na_2_, and 10 μM PMSF). The homogenate was centrifuged at 41,000 g for 1 h at 4 °C. The filtered supernatant served as the crude enzyme source.

Step 2: Chromatography on a Sephadex G-25. All the collected supernatant from step 1 was put on a Sephadex G-25 column. The portions were gathered at a continuous flow rate of 40 mL/h at 4 °C.

Step 3: Procainamide affinity chromatography and removal of procainamide and condensation. The gathered AChE sample from step 2 was loaded on the procainamide-based Sepharose 4B affinity column. The affinity column was eluted with phosphate buffer (0.1 M, pH 8.0, containing 0.2 M Nalco) and phosphate buffer (0.1 M, pH 8.0, containing 0.5 M Nalco) was used to remove protein impurity. Then the AChE was eluted with Net_4_I (0.05 M) in phosphate buffer (0.1 M, pH 8.0, containing 0.5 M Nalco). The collected AChE was dialyzed against phosphate buffer (0.05 M, pH 8.0) three times at 4 °C for 3 h. The purified enzyme can be stored at −80 °C and served as the enzyme source.

#### Measurement of AChE activity

AChE activity was measured following the method of Gorun *et al*.^41^. Reaction mixtures contained 0.1 mL ATChI and 0.1 mL purified enzyme source. The reaction was started when the enzyme was added. Subsequently, the reaction mixtures were incubated at 30 °C for 15 min. The reaction was stopped by adding 3.6 mL DTNB. The AChE activity was determined by measuring the optical density (OD) of the reaction product at 412 nm^40^. Each treatment was replicated three times. Enzyme was added to the control groups after the reaction stopped. Protein concentration was measured by the method of Bradford^42^ and BSA was used as the standard.

#### Determination of AChE bimolecular rate constant K_i_

The K_i_ values were measured for each enzyme by incubation with the inhibitor in assay buffer and by assaying aliquots for remaining AChE activity at various time points using SigmaPlot (Systat Software, USA).

### Sequence analysis and single nucleotide polymorphism (SNP) detection of *Mdace*

Total RNA was extracted from each female housefly in the PSS and N-PRS strains, with RN07-EASYspin Tissue/Cellular RNA Rapid Extraction Kit (Aid lab, Beijing, China). The first-strand cDNA was synthesized using Prime Script™ 1st Strand cDNA Synthesis Kit (Takara Biotechnology, Dalian, China). The full length of *Mdace* cDNA was amplified using primer pair 1 (forward: 5′TCAACATCCAATCCATATCCCAG3′; reverse: 5′ATGTGTATGTGTGTGGGTGAGTGTT3′). The 20 μL PCR reaction mixture contained 1.0 μL cDNA, 0.5 μL AChE-F primer (10 μM), 0.5 μL AChE-R primer (10 μM), 10 μL 2 × Taq Master Mix (Dye Plus), and 8 μl ddH_2_O (Vazyme, Nanjing, China). The PCR program: an initial denaturation step of 94 °C for 5 min, followed by 35 cycles of PCR reaction (94 °C for 30 sec, 55 °C for 30 sec, and 72 °C for 2.5 min) and a final extension of 72 °C for 10 min. The PCR product was directly sequenced by Beijing TSINGKE Biological Technology. The sequences were aligned using DNAMAN (Lynnon Biosoft, USA) and the polymorphisms of mutations were analyzed by the chromatogram viewer Chromas (Technelysium Pty Ltd). A total of 60 individual houseflies were utilized, with 30 for each population.

### Real-time quantitative PCR

Total RNA and genomic DNA were extracted from heads of PSS and N-PRS females to test the expression levels of mRNA and DNA of the *Mdace* gene by quantitative real-time PCR^43^. The reactions were performed on Applied Biosystems 7500 Real-time PCR system (Applied Biosystems, Foster City, CA, USA). The primer pair 2 (forward:5′ATCACCTGGCCGCTAGAAAC′; reverse:5′TCGCGACCCTGAACTGTAAC3′) was designed for *Mdace* based on the gene sequence. The *GAPDH* gene was served as internal reference gene^44^. The standard curve of *Mdace* and the internal reference gene *GAPDH* were done by the threshold cycle of a serial 2-fold dilution of cDNA. The reactions were conducted in 20 μL containing 1 μL cDNA (1 μg in total) or 4 μL DNA (100 ng in total) template, 10 μL 2×SYBR Premix Ex Taq (Takara), 0.4 μL forward primer (10 mM), 0.4 μL reverse primer (10 mM), 0.4 μL Rox II (Takara), and 7.8 μL (cDNA) or 4.8 μL (DNA) nuclease-free water. The qRT-PCR program was as follows: 95 °C for 2 min, followed by 40 cycles of PCR reaction (95 °C for 15 sec, 60 °C for 30 sec). After amplification, a final dissociation stage (95 °C for 15 sec, 60 °C for 15 sec, and 95 °C for 15 sec) was performed to ensure the amplification product was specific. Three technical replications and three biological replications were conducted in our study. qRT-PCR data was analyzed using the 2^-△△Ct^ method^45^.

## Data analysis

The relative mRNA expression level and DNA copy number of *Mdace* in the PSS and N-PRS strains were determined by an *t*-test using the GraphPad InStat 3.0 software (GraphPad Software, San Diego, CA, USA).
